# Rituximab in the treatment of progressive interstitial lung disease associated with the antisynthetase syndrome

**DOI:** 10.1186/s13075-024-03353-2

**Published:** 2024-06-18

**Authors:** Javier Narváez, Elena Cañadillas, Iván Castellví, Juan José Alegre, Vanesa Vicens-Zygmunt, Guadalupe Bermudo, Paola Vidal-Montal, María Molina Molina, Joan Miquel Nolla

**Affiliations:** 1 Department of Rheumatology, Hospital Universitario de Bellvitge. Bellvitge Biomedical Research Institute (IDIBELL), Feixa Llarga, s/n, Hospitalet de Llobregat, 08907 Barcelona, Spain; 2 Department of Rheumatology, Hospital Clínico, Universitario Lozano Blesa, Zaragoza, Spain; 3 Department of Rheumatology. Hospital, Universitario de la Santa Creu i Sant Pau, Barcelona, Spain; 4 Department of Rheumatology. Hospital, Universitario Dr Peset, Valencia, Spain; 5 Interstitial Lung Disease Unit, Department of Pneumology. Hospital, Universitario de Bellvitge. Bellvitge Biomedical Research Institute (IDIBELL), Barcelona, Spain

**Keywords:** Antisynthetase syndrome; Progressive interstitial lung disease; Treatment; Rituximab

## Abstract

**Objective:**

To assess the real-world, long-term effectiveness of rituximab (RTX) as a rescue therapy in patients with antisynthetase syndrome and progressive interstitial lung disease (ASS-ILD).

**Methods:**

Multicentre observational retrospective longitudinal study of a cohort of patients with ASS-ILD that started treatment with RTX due to recurrent or ongoing progressive ILD despite therapy with glucocorticoids and immunosuppressants.

**Results:**

Twenty-eight patients were analyzed. Examining the entire study population, before treatment with RTX the mean decline in %pFVC and %pDLCO from the ASS-ILD diagnosis to the initiation of RTX treatment (T0) was -6.44% and -14.85%, respectively. After six months of treatment, RTX reversed the decline in pulmonary function test (PFT) parameters: ∆%pFVC +6.29% (95% CI: -10.07 to 2.51; *p*=0.002 compared to T0) and ∆%pDLCO +6.15% (95% CI: -10.86 to -1.43; *p*=0.013).

Twenty-four patients completed one year of therapy and 22 two years, maintaining the response in PFT: ∆%pFVC: +9.93% (95% CI: -15.61 to -4.25; *p*=0.002) and ∆%pDLCO: +7.66% (95% CI: -11.67 to -3.65; *p*<0.001). In addition, there was a significant reduction in the median dose of prednisone, and it could be suspended in 18% of cases. In 33% of patients who required oxygen therapy at the start of treatment, it could be discontinued.

The frequency of adverse events reached 28.5% of cases.

**Conclusion:**

Based on our results, RTX appears to be effective as rescue therapy in most patients with recurrent or progressive ASS-ILD unresponsive to conventional treatment. The use of RTX was well tolerated in the majority of patients.

## Background

Antisynthetase syndrome (ASS) is a systemic autoimmune condition characterized by the positivity of anti-aminoacyl-transfer-RNA synthetases antibodies (ARS) with clinical features that may include myositis, arthritis, interstitial lung disease (ILD), Raynaud’s phenomenon, fever and mechanic’s hands, which are the six hallmark symptoms of the disease.

ILD is the most severe complication of ASS, with a prevalence ranging from 51 to 100%, higher than those rates reported in dermatomyositis (DM) and polymyositis, with which it shares many features [1–5]. In addition to its frequency, ILD is the leading cause of morbidity and mortality for this condition, causing an excess 5-year mortality of up to 45% [1–5].

There is no standardized approach to the treatment of ASS-ILD due to the absence of randomized controlled trials comparing various agents specifically available for this disorder. Glucocorticoids (GC) at medium or high doses are the first line of treatment. Several conventional immunosuppressants (IS) have been used in conjunction with GC, either if the patient fails to improve or since the beginning of the treatment in patients with severe clinical and functional decline. However, disease progression over time remains relevant in 32–35% of cases among patients treated with steroids in association with IS [1–5]. Therefore, rescue therapy is needed for this patient subgroup who barely respond to GC and IS.

Rituximab (RTX) seems to be a promising candidate. Throughout the last decade, several studies have assessed the effects of RTX on the treatment of connective tissue disease-related interstitial lung disease (CTDs-ILD) with mostly promising results [6–9], including two randomized, controlled phase II clinical trials for systemic sclerosis [10, 11]. In four recent observational studies, RTX appeared capable of stabilizing or improving ILD in patients with ASS [12–15].

This study aimed to evaluate the long-term effectiveness and safety of RTX as a rescue therapy in a real-world cohort of patients with recurrent or ongoing progressive ASS-ILD despite conventional treatment.

## Methods

### Study sample

We conducted a review of medical records and hospital pharmacy-prescribing databases, identifying all ASS patients who started RTX administered as part of routine clinical care at three referral tertiary care hospitals. From this registry, we selected for analysis all consecutive adult patients with recurrent or ongoing progressive ASS-ILD despite treatment with GC and IS, treated with at least 1 cycle of RTX and evaluated via pulmonary function tests (PFT) for at least 6 months after RTX treatment. No patients were excluded due to poor outcomes or early death.

Informed consent was obtained from the patients and their clinical records and information were anonymized prior to analysis.

As with other previous studies from our group [7, 8], progressive ILD was stated when there was a relative decline of ≥10% in the predicted forced vital capacity (%pFVC) or ≥15% in the predicted diffusing capacity for carbon monoxide corrected for hemoglobin (%pDLCO) over 24 months despite treatment or a relative decline in the %pFVC of 5–10% or less than 15% in the %pDLCO, as well as a worsening of respiratory symptoms and increased fibrosis as determined by thoracic high-resolution computed tomography (HRCT). Half of patients (15/28) fulfilled the 2022 criteria of progressive pulmonary fibrosis (PPF).

ASS was diagnosed according to the Solomon 2011 diagnostic criteria [16]. ILD was diagnosed by HRCT of the chest, as determined by experienced thoracic radiologists who classified cases into 3 general radiologic patterns according to the American Thoracic Society (ATS)/European Respiratory Society (ERS) International Multidisciplinary Consensus Classification of Idiopathic Interstitial Pneumonias [17]: 1) usual interstitial pneumonia (UIP); 2) nonspecific interstitial pneumonia (NSIP); and 3) organizing pneumonia (OP) or NSIP superimposed with OP. In all cases, the conclusive diagnosis of ASS-ILD was formulated in a multidisciplinary context.

### Treatment

RTX therapy consisted of 2 intravenous (IV) infusions of 1 g per treatment cycle separated by a 2-week interval (days 1 and 15). Treatment was repeated after at least 6 months, depending on the pulmonary response and immunoglobulin levels.

Patients were followed at a special multidisciplinary unit by both a pneumologist and a rheumatologist. Ongoing therapy with IS and GC remained initially unchanged in all cases, although increased or decreased doses of prednisone were possible after starting RTX at the discretion of the treating physician.

### Clinical assessments and outcome variables

The efficacy of RTX was evaluated according to the following measurements: 1) the changes in the %pFVC and %pDLCO before and after start of therapy with RTX; 2) the distance covered in the 6-minute walking test (6MWT); and 3) changes in the post-treatment HRCT.

After the start of treatment with RTX, PFT and the 6MWT were performed every 6 months. PFT were undertaken in a standardized manner in all patients based on the 2002 recommendations of the Spanish Society of Pneumology and Thoracic Surgery [18]. The %pFVC and %pDLCO were both performed at the same time in each patient.

The evolution of PFTs was classified according to the definitions of the ATS into worsening (a decrease of %pFVC >10% or %pDLCO >15%), stabilization (if changes in %pFVC were less than 10% or 15% in %pDLCO), or improvement (increase of %pFVC >10% or %pDLCO >15%) [19, 20].

Post-treatment HRCT scans were obtained of patients with worsening dyspnea and/or deterioration in lung function or to evaluate the response to treatment in those who gave their consent.

ARS were measured using the EUROLINE Autoimmune Inflammatory Myopathies Profile (EUROIMMUN, Germany).

Information about RTX therapy included the number of received cycles, doses administered, follow-up duration from the first dose, continuation or discontinuation of the treatment at the endpoint of patient follow-up, reason for discontinuation (if any), and the tolerability and side effects profile (including infusion reactions, hypogammaglobulinemia, neutropenia and infections). In case of death, we analyzed the relationship between the death and RTX. The endpoint of patient follow-up was the date of the last clinic visit. A retrospective analysis of prospectively collected data was performed.

### Statistical analysis

Results are expressed as the mean ± standard deviation (SD) or as the median (interquartile range [IQR], 25th-75th) as appropriate for continuous data, while categorical variables are presented as the number of cases and percentages.

The Student t or Mann–Whitney U test was used to compare numerical variables according to normality adjustments and the chi-squared or Fisher’s exact test was used for categorical variables.

Pulmonary function trends were quantified as a percentage change (delta) from the diagnosis of ASS-ILD to the initiation of RTX treatment (T0), and in relation to T0 for all subsequent evaluations after starting RTX therapy. The paired sample t-test was used to compare pre- and post-RTX means of the main outcome efficacy measures evaluated. Statistical significance was defined as *P* < 0.05.

## Results

### Patient characteristics

To date, we have treated 28 patients with RTX for recurrent (N=1) or ongoing progressive ASS-ILD (N=27) despite having been previously administered GC and IS.

The main baseline characteristics of this population are summarized in Table 1. The mean (± SD) age at RTX onset was 61±15 years. At that time, the median duration of ILD was 15 months (interquartile range [IQR]25th–75th: 12–21 months).

**Table 1:** Baseline characteristics of the 28 patients with progressive antisynthetase syndrome-related interstitial lung disease (ASS-ILD) treated with Rituximab

Number of patients	28
Age (mean ± SD), years	61 ± 15
Women/men (ratio)	20 (71.5%) / 8 (28.5%)
Median duration of ILD, months [IQR 25th–75th]	15 (12 – 21)
**Frequency of clinical features**	
Fever	9 (32%)
Raynaud's phenomenon	13 (46%)
Myositis	21 (75%)
Arthritis	15 (54%)
Mechanic’s hands	18 (64%)
Hiker’s foot	3 (11%)
Gottron’s sign	5 (18%)
Periungual erythema	5 (18%)
Interstitial lung disease (ILD)	28 (100%)
Chest HRCT pattern of ILD	
* Non-specific interstitial pneumonia (*NSIP)	19 (68%)
* Organizing pneumonia (OP) or NSIP superimposed with OP*	6 (21%)
* Usual interstitial pneumonia*	3 (11%)
%FVC predicted at ILD diagnosis, mean ± SD (I*QR* 25%-75%)	76.3 ± 21.6 (57.9 – 87.8)
%DLCO predicted at ILD diagnosis, mean ± SD (I*QR* 25%-75%)	58,3 ± 16,9 (43 – 70)
**Serological parameters**	
Positive antinuclear antibodies (ANA)	22 (79%)
* Myositis-specific antibody, n (%)*	
* Anti-Jo-1*	17 (61%)
* Anti-PL-7*	4 (14%)
* Anti-PL-12*	4 (14%)
* Anti-EJ*	3 (11%)
* Myositis-associated antibody, n (%)*	
Anti-Ro52	18 (64%)

*HRCT* thoracic high-resolution computed tomography, *%pFVC* predicted forced vital capacity, *%pDLCO* predicted diffusing capacity for carbon monoxide, corrected for hemoglobin

Anti-Jo-1 antibodies were the commonest ARS (61%), followed by anti-PL-7 (14%) and anti-PL-12 (14%). According to the radiological diagnosis, 19 (68%) cases corresponded to NSIP (12 with a fibrotic subtype), 6 (21%) to OP or NSIP superimposed with OP, and the remaining 3 (11%) to UIP.

At the time of RTX onset, the mean %pFVC was 69.8±19.6 (IQR 52.5–76.7), the mean %pDLCO was 43.4±14.6 (IQR 32–52), and the mean distance covered in the 6MWT was 343±112 m (IQR 289–442).

### Treatment characteristics

Before treatment with RTX (see Table 2), all patients had previously been treated with high-medium prednisone doses, and one or more IS, including mycophenolate [MMF] (71%), azathioprine [AZA] (46%), tacrolimus [TAC] (46%) or cyclosporine A [CsA] (11%), intravenous cyclophosphamide [CYC] (11%), methotrexate (14%) and/or leflunomide (4%). All patients treated initially with CYC (500–750 mg/m2 every month for six doses) were later transitioned to MMF or another IS. In all cases, the time elapsed since the last dose of CYC was greater than one year. Seven patients (25%) had also undergone treatment with intravenous immunoglobulin at doses of 400 mg/kg daily for five days for at least 3 months.

**Table 2:** Previous and concomitant treatments of the 28 patients with progressive ASS-ILD treated with Rituximab

Need for intravenous methylprednisolone boluses	10 (36%)
Number of prior immunosuppressants used (mean)	2 (range, 1-5)
*Prior treatment with Mycophenolate*	*20 (71%)*
*Prior treatment with Azathioprine*	*13 (46%)*
*Prior treatment with Cyclophosphamide*	*3 (11%)*
*Prior treatment with Tacrolimus / Cyclosporine A*	*13 (46%) / 3 (11%)*
*Prior treatment with Methotrexate*	*4 (14%)*
*Prior treatment with Leflunomide*	*1 (4%)*
*Prior treatment with Hydroxychloroquine*	*3 (11%)*
Need for intravenous immunoglobulins	7 (25%)
Rituximab Monotherapy, n (%)	1 (4%)
RTX plus a concomitant immunosupressant, n (%)	27 (96%)
Mycophenolate	13 (46%)
Tacrolimus	10 (36%)
Azathioprine	3 (11%)
Ciclosporine A	1 (4%)
Mean dose of prednisone at RTX initiation, mg/d [IQR 25th–75th]	19 (5, 30)
Need for oxygen theratpy at RTX initiation	12 (43%)
Need for antifibrotic therapy	3 (11%)
%pFVC at first RTX infusion, mean ± SD [IQR 25th–75th]	69.8 ± 19.6 (52.5 – 76.7)
%pDLCO at first RTX at first RTX infusion, mean ± SD [IQR 25th–75th]	43.4 ± 14.6 (32 – 52)

*%pFVC* predicted forced vital capacity, *%pDLCO* predicted diffusing capacity for carbon monoxide, corrected for hemoglobin, *RTX* Rituximab

Of the 28 patients, 1 (4%) received RTX monotherapy, while 27 (96%) received RTX plus concomitant IS (Table 2): 13 (46%) MMF (at a dose of 1.5-2 g/d), 10 (36%) TAC (administered at doses to achieve plasma levels between 5 to 15 ng/mL), 3 (11%) AZA (at a dose of 100 mg/d), and 1 (4%) CsA (3 mg/kg/day). Ongoing therapy with hydroxychloroquine (HCQ) for skin manifestations was maintained in two patients and with antifibrotic agents in three (2 nintedanib and 1 pirfenidone).

The mean dose of prednisone taken by the patients at the time of RTX initiation was 19 mg/day [IQR 25th–75th: 5–30]. Twelve patients (43%) required oxygen therapy at RTX onset

The number of RTX cycles administered (mean ± SD) was 4 ± 2.6 (range, 1-11), and the median time of follow-up after RTX treatment was 48 months (IQR 24-86). The total follow-up was 135.6 patient-years.

### Efficacy endpoints

**Fig. 1:**
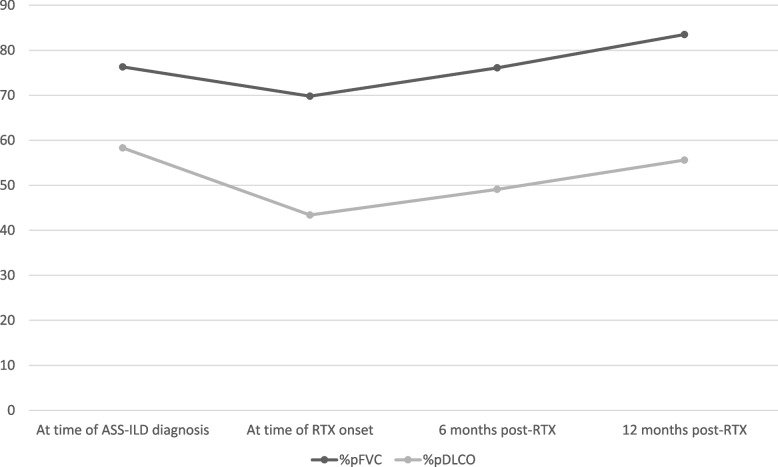
Evolution of the predicted forced vital capacity (%pFVC) and the predicted diffusing capacity for carbon monoxide corrected for haemoglobin (%pDLCO) before initiation of RTX and after 1 year of treatment

Changes in the main outcome efficacy measures evaluated before and after 6 months, 1 and 2 years of treatment with RTX are detailed in Tables 3 and 4 and Fig. 1. Considering the entire study population, prior to initiation of RTX the mean decline in %pFVC and %pDLCO from the ILD diagnosis to the initiation of RTX treatment (T0) was -6.44% (95% CI: 2.45 to 10.43; p=0.003) and -14.85% (95% CI: 10.51 to 19.19; p<0.001), respectively. After 6 months of treatment, RTX proved capable of reversing the decline in PFT parameters (delta: percentage change from the start of therapy): ∆%pFVC +6.29% (95% CI: -10.07 to 2.51; p=0.002 compared to T0) and ∆%pDLCO +6.15% (95% CI: -10.86 to -1.43; *p*=0.013).

**Table 3:** Changes before and after 6 months, 1 and 2 years of treatment with Rituximab vis-à-vis the main outcome efficacy measures evaluated

Before RTX treatment				
	At time of ASS-ILD diagnosis mean ± SD (IQR, 25th-75^th^)	At time of RTX onset mean ± SD (IQR, 25th-75^th^)	Delta (mean)	*P* (95% CI)
Total sample (*N*=28)				
%FVC predicted	76.3 ± 21.6 (57.9 – 87.8)	69.8 ± 19.6 (52.5 – 76.7)	-6.44%	0.003 (2.45 to 10.43)
%DLCO predicted	58.3 ± 16.9 (43 – 70)	43.4 ± 14.6 (32 – 52)	-14.85%	<0.001 (10.51 to 19.19)
Mean distance covered in 6MWT (meter)	420 ± 88 (349 – 490)	343 ± 112 (289.5 – 44.5)	-77.68 m	0.021 (13.50 to 141.87)
After 6 months of treatment				
	At time of RTX onset mean ± SD (IQR, 25th-75^th^)	6 months post-RTX mean ± SD (IQR, 25th-75^th^)	Delta (mean)	*P* (95% CI)
Total sample (*N*=28)				
%FVC predicted	69.8 ± 19.6 (52.5 – 76.7)	76.1 ± 21.6 (60.7 – 90.9)	+6.29%	0.002 (-10.07 to -2.51)
%DLCO predicted	43.1 ± 14.8 (32 – 52)	49.1 ± 15.8 (35.7 – 55)	+6.15%	0.013 (-10.86 to -1.43)
Mean distance covered in 6MWT (meter)	343 ± 112 (289.5 – 442.5)	433 ± 76 (382.5 – 488)	+90.41 m	0.018 (-197.95 to -22.87)
After 1 year of treatment				
	At time of RTX onset mean ± SD (IQR, 25th-75^th^)	12 months post-RTX mean ± SD (IQR, 25th-75^th^)	Delta (mean)	*P* (95% CI)
Total sample (*N*=24)				
%FVC predicted	71.9 ± 20.1 (57 – 80)	83.5 ± 22 (66.7 – 102.5)	+11.63%	<0.001 (-16.91 to -6.36)
%DLCO predicted	45.3 ± 16 (32 – 52.7)	55.6 ± 15.7 (44 – 63)	+10.29%	<0.001 (-14.82 to -5.76)
Mean distance covered in 6MWT (meter)	346 ± 124 (290 – 450)	433.4 ± 73 (361.5 – 471)	+87.16 m	0.061 (-179.21 to 4.88)
After 2 years of treatment				
	At time of RTX onset mean ± SD (IQR, 25th-75^th^)	24 months post-RTX mean ± SD (IQR, 25th-75^th^)	Delta (mean)	*P* (95% CI)
Total sample (*N*=22)				
%FVC predicted	74.1 ± 19.5 (60.3 – 82)	84 ± 21.6 (69.9 – 99.2)	+9.93%	0.002 (-15.61 to -4.25)
%DLCO predicted	45.5 ± 14.7 (32 – 54)	53.2 ± 16.8 (43.7 – 62.6)	+7.66%	<0.001 (-11,67 to -3,65)
Mean distance covered in 6MWT (meter)	334 ± 136 (289 – 456)	438 ± 91.5 (367.5 – 528.5)	+103.54 m	0.33 (-197.17 to -9.91)

*%pFVC* predicted forced vital capacity, *%pDLCO* predicted diffusing capacity for carbon monoxide, corrected for hemoglobin, *RTX* Rituximab, *6MWT* 6-minute walking test

**Table 4:**
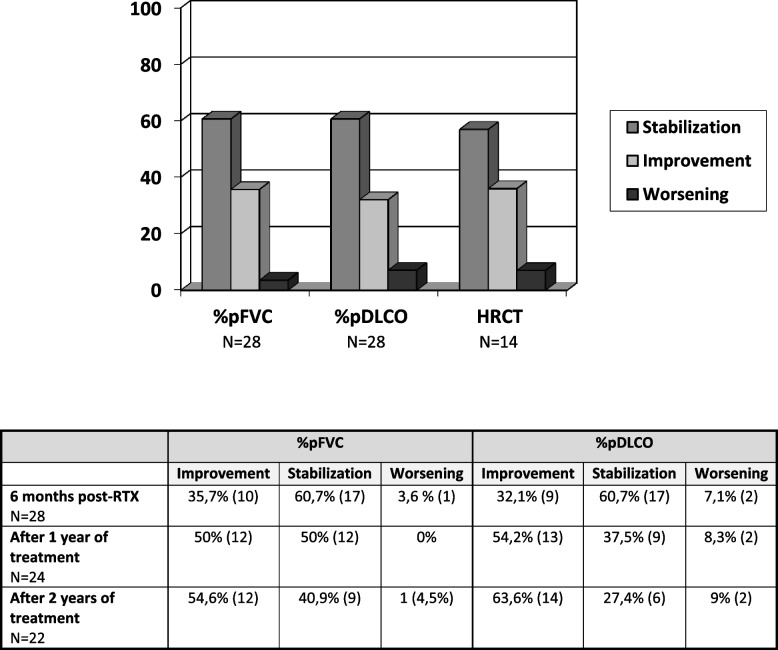
Lung function tests results (according to the definitions of the ATS) and response of the ILD radiologic features after Rituximab therapy

*%pFVC* predicted forced vital capacity, *%pDLCO* predicted diffusing capacity for carbon monoxide, corrected for hemoglobin, *RTX* Rituximab

At 1 year of treatment, no data was available for four patients: one died at 6 months due to the progression of the ILD, and three had not yet completed 12 months of treatment. In the 24 patients that completed one year of therapy, a significant improvement in %pFVC (∆+11.63%, 95% CI: -16.91 to -6.36; *p*<0.001) and %pDLCO (∆+10.29%, 95% CI: -14.82 to -5.76; *p*<0.001) was observed (see Fig. 1), as well as an increase in the distance covered in the 6MWT (∆ +87 m, 95% CI: -179.21 to -4.88; *p*=0.061). In addition, the average dose of prednisone was reduced to 4.5 mg/d (Delta –14.5 mg/d; 95% CI: 7.73 to 15.62, *p*<0.001), and could be stopped in 5 (18%) patients.

Comparing pre- and post-RTX pulmonary variables, %pFVC and %pDLCO were stable or improved in 92% of subjects at 6 months according to the ATS definitions, and in 88% at 12 months (refer to Table 4). Results stratified by myositis-specific autoantibody positivity and HRCT ILD patterns of interstitial lung disease are presented in Table 5.

**Table 5:** Results stratified by myositis-specific autoantibody positivity and high-resolution computed tomography (HRCT) patterns of interstitial lung disease

	**6 months post-RTX** ***N*=28**		**1-year post-RTX** ***N*=24**	
	Improvement or stabilization of %pFVC and %pDLCO	Worsening of %pFVC and/or %pDLCO or death	Improvement or stabilization of %pFVC and %pDLCO	Worsening of %pFVC and/or %pDLCO or death
**Chest HRCT pattern of ILD**				
Non-specific interstitial pneumonia (NSIP)	94.7% (18/19)	5.3% (1/19)	93.8% (15/16)	6.2% (1/16)
Organizing pneumonia (OP) or NSIP superimposed with OP	100% (6/6)	0% (0/6)	83.3% (5/6)	16.7% (1/6)
Usual interstitial pneumonia (UIP)	50% (1/2)	50% (1/2)	100% (2/2)	0% (0/2)
***Myositis-specific antibody***				
Anti-Jo-1	88.2% (15/17)	11.8% (2/17)	85.7% (12/14)	14.3% (2/14)
Anti-PL-7	100% (4/4)	0% (0/4)	100% (4/4)	0% (0/4)
Anti-PL-12	100% (4/4)	0% (0/4)	100% (3/3)	0% (0/3)
Anti-EJ	100% (3/3)	0% (0/3)	100% (3/3)	0% (0/3)

Comparing pre- and post-RTX pulmonary variables, %pFVC and %pDLCO were stable or improved in 92% of subjects at 6 months according to the ATS definitions (see Table 4). Results stratified by myositis-specific autoantibody positivity and HRCT ILD pattern are shown in Table 5.

Twenty-two of the 24 patients completed 2 years of treatment (two had not yet completed 24 months of treatment at the last visit), maintaining the response in pulmonary function tests: ∆%pFVC: +9,93% (95% CI: -15.61 to -4.25; p=0.002) and ∆%pDLCO: +7.66% (95% CI: -11.67 to -3.65; p<0.001). Four of the 12 patients (33%) who required oxygen therapy at the start of treatment were able to discontinue it within the first 2 years of treatment with RTX.

Post-treatment HRCT scans were available in 14 (50%) patients: two were treated with RTX for 1 year (2 cycles), and 12 for ≥ 2 years. The median time between RTX initiation and the HRCT control was 19 months (IQR: 13-28). HRCT thorax images were scored for ground-glass attenuation, fibrosis (including thickened reticular markings, bronchiectasis and bronchiolectasis) and honeycombing. Estimation of the extent and severity of ILD radiologic features was based on visual assessment and not using a quantitative computer-based CT algorithm. In one (7%) patient the ILD radiologic features worsened, in five (36%) they improved, and in eight (57%) they remained stable.

At the end of the follow-up period, only 18 of the 28 patients (64.3%) were still undergoing treatment with RTX: one (3.5%) died at 6 months, in three cases (11%) the treatment was stopped due to adverse events (severe infusional reaction in 1 case and repeated infections in 2), in one patient (3.5%) treatment failed, and in five patients (18%) RTX was stopped due to marked clinical improvement.

Considering only the subgroup of patients that continued treatment beyond 24 months for the efficacy analyses (*N*=18), the change in %pFVC at the end of follow-up (median 67 months; IQ 52–116) was +14.91% (95% CI -21.86 to -7.96, *p*<0.001 compared to baseline) and +16.61 in %pDLCO (95% CI -24.76 to -8.45, *p*=0.002).

### Safety and survival

The frequency of adverse events reached 28.5% of cases (8/28), resulting in the withdrawal of RTX in the three patients mentioned above (11%). The side effects included respiratory infections (14%), with 1 case of SARS-COV-2 pneumonia, one case of herpes zoster infection, one case of cytomegalovirus colitis, one severe infusional reaction, IgG and/or IgM hypogammaglobulinemia (18%), and transient mild-moderate neutropenia (7%).

## Discussion

Observational studies have suggested that progressive ILD is the major determinant of morbidity and mortality in ASS [1–5]. Among the most urgent challenges facing clinicians is how to best determine which drugs can be useful as a rescue treatment in patients who do not respond to conventional therapy with GC and IS. In this sense, RTX appears to be one of the most promising candidates. Since testing RTX in ASS in a randomized controlled trial would be quite challenging, observational data can be informative.

Based on our real-world experience, RTX rescue therapy effectively reversed the decline of lung function parameters in a significant proportion of patients with recurrent or ongoing progressive ASS-ILD who had not responded to conventional therapy, achieving stabilization or amelioration of these variables (%pFVC and %pDLCO), as well as HRCT findings. Additionally, there was a significant reduction in the median dose of prednisone in the months following RTX initiation, which could be stopped in 18% of patients. Furthermore, 33% of patients were able to stop oxygen therapy. Of interest, fifteen (53.6%) of our patients met the 2022 criteria for progressive pulmonary fibrosis (PPF) [21], fulfilling at least two of the following three criteria: worsening respiratory symptoms, physiological progression (an absolute decline in %pFVC of ≥5% predicted and/or an absolute decline in %pDLCO of ≥10% within 1 year of follow-up), and radiological progression. With currently available treatments, the most realistic goal in patients with established ongoing progressive ASS-ILD is to slow down or stop disease progression, particularly in those with PPF. From this perspective, the stabilization of lung disease can be regarded as a success, especially when it is achieved while the patient still retains adequate functional capacity and does not need domiciliary oxygen therapy.

Our results are concordant with the increasing evidence published in the literature supporting the beneficial effects of RTX in ASS-ILD, although this is limited to case reports and series, as well as a few observational studies. The main findings of the four previously published studies that have evaluated RTX's efficacy and safety in ASS-ILD are summarized in Table 6.

**Table 6:** Main characteristics of the previous studies that have evaluated the efficacy and safety of Rituximab in the treatment of antisynthetase syndrome-related interstitial lung disease (AS-ILD)

**Study (reference)**	**No. pts**	**Type of study**	**Refractory manifestations**	**RTX regimen**	**Follow up**	**Outcomes**	**Safety of RTX**
Andersson et al. [12]	34	Retrospective	Progressive ILD (70.5%). Two-thirds of the patients also had signs of myositis, with elevated CK levels and/or reduced MMT8 scores	RA scheme	52 m	Improvement in PFT (CVF increased by 24% and DLCO by 17%) and HRCT (the extent of ILD in HRCT scans decreased by 34%) post-RTX. MMT8 score increased from a median 93% of the maximum score pre-RTX to a median 98% post-RTX (P<0.05)	Serious infection: 18% Mortality: 21% The mortality rate in the RTX-treated group was comparable to that of the remaining ASS cohort
Doyle TJ et al. [13]	25	Retrospective	Recurrent or progressive ILD (84%) Myositis (90.4%) Mechanic’s hands/rashes/arthritis (57%)	RA scheme	1 to 3 yrs	Stability or improvement in PFT (FVC, TLC and DLCO) and HRCT in 88% and 79% of subjects, respectively. Further, there was a significant steroid-sparing effect (GC dose decreased from 18 ± 9 to 12 ± 12 mg/day)	No serious AE
Langlois et al. [14]	28 RTX *vs*. 32 CYC	Retrospective	ILD (100%) Muscle weakness (71%) Arthralgia/arthritis (76%) Cutaneous involvement (55%)	RA scheme	2 yrs	Improved PFTs and HRCT score in both groups. RTX and CYC demonstrated similar pulmonary progression-free survival (PFS) at six months after treatment. However, RTX proved superior to CYC at 2 years of treatment (HR 0.263)	Similar AE
Allenbach et al. [15]	10	Prospective Open label, phase II trial	10 patients with refractory ASS Myositis (100%) ILD (100%)	RA scheme	1 year	Improvement of PFT in 5 patients, stability in 4, and worsening in 1 Stable HRCT score 8 of 10 patients showed muscular improvement on MMT10 and normalization of creatine kinase levels The steroid dose was decreased in 6 patients	No serious AE

*CYC* Cyclophosphamide, *GC* Glucocorticoids, *HRCT* thoracic high-resolution computed tomography, *ILD* Interstitial lung disease, *MMT* Manual muscle testing, *%pFVC* predicted forced vital capacity, *%pDLCO* predicted diffusing capacity for carbon monoxide, corrected for hemoglobin, *PFT* Pulmonary function tests, *RA* Rheumatoid arthritis, RTX Rituximab

According to the study by Andersson et al. [12] involving 24 patients with severe refractory ASS-ILD, the median percentages of predicted FVC and DLCO increased by 24% and 17%, respectively, while the extent of ILD in HRCT scans decreased by 34% after RTX therapy (median follow-up post-RTX of 52 months). There were seven deaths among the RTX-treated patients, six of which were probably caused by infection. Although infections were the main cause of death in this study, the overall mortality rate was comparable in the RTX-treated and non-RTX-treated patients. Of note, 10 out of 12 patients with acute onset or exacerbation were on combined therapy with RTX and CYC. Combining two immunosuppressive agents could have aggravated the risk of fatal infections; therefore, the high infection rate cannot be attributed solely to the use of RTX.

In another US multicentric retrospective study involving 25 patients with recurrent or progressive ASS-ILD, RTX led to stabilization or improvement in the HRCT score and %FVC in 88% and 79% of patients, respectively, after one year of treatment [13]. An increase in total lung capacity was noted, as well as an improvement in %DLCO after three years of follow-up, suggesting a potential benefit of retreatment. In this sense, subgroup analyses demonstrated the benefits of repeating RTX dosing over a single RTX cycle, with the greatest benefit from RTX observed at the 3-year follow-up mark. The addition of RTX enabled a tapering off the prednisone dose by almost half in 88% of cases. The use of RTX was well tolerated by the majority of patients. In this study, twenty-one out of 25 patients were treated with RTX as a second-line medication because of unresponsiveness to previous GC and IS, and 4 out of 25 were administered RTX as a first-line treatment.

Langlois et al. [14] compared CYC (n=32) followed by standard IS (AZA, MMF, MTX) vs. RTX (n=28) administered every 6 months in patients with ASS-ILD. RTX and CYC demonstrated similar pulmonary progression-free survival (PFS) at six months after treatment (92% RTX vs. 85% CYC, respectively). However, RTX proved superior to CYC at 2 years of treatment (HR 0.263, 95% CI 0.094–0.732, *p*=0.011) despite the fact that it was administered to patients with more refractory disease compared to those cases in which CYC was given. RTX treatment also proved superior during the maintenance phase compared with IS continued after CYC. No significant differences were found either in the steroid-sparing effect or in the frequency of adverse effects.

Only one small prospective study has been published, which involved 10 patients presenting refractory ASS treated with two cycles of RTX [15]. Refractory disease was defined as intolerance or inadequate response to GLC and at least two IS agents. In this trial, 8 of 10 patients showed muscular improvement based on manual muscular testing (MMT10, Kendall score in 10 muscles) and normalization of creatine kinase levels, and 9 of 10 patients exhibited improved or stabilized lung function at the 1-year follow-up visit. Moreover, the steroid dose was decreased in 6 patients, or they were discontinued from other IS drugs. In only one case, the authors described a reduction in interstitial infiltrates on HRCT. There were six infectious adverse events, none of them severe.

Regarding the published case reports and case series, we identified 20 reports that describe a total of 65 patients with ASS-ILD [22–41]. Stabilization or significant improvement in ILD was reported in most cases, while there was only one mortality linked to *Pneumocystis jirovecii* pneumonia [38].

A better response to RTX has been described in patients with DM and ASS compared to other forms of CTDs [42, 43]. This was confirmed in a recent meta-analysis that analyzed the use of RTX for the treatment of CTD-ILD and found that patients with AAS-ILD and idiopathic inflammatory myopathies associated with ILD had a higher improvement rate compared to other types of CTD-ILD, including rheumatoid arthritis, systemic sclerosis, mixed connective tissue disease (MCTD) and undifferentiated connective tissue-disease (UTCD). The pooled improvement rate for ASS-ILD was 48.1% (95% CI: 0.373-0.620) and for IIM-ILD non-ASS, it was 47.4% (95% CI: 0.266-0.846) [44].

When interpreting the results of our study, one must acknowledge potential limitations inherent to its retrospective observational nature, the small sample size, the selection bias (patients were all worsening prior to starting RTX, which may bias the results towards improvement/stabilization after this time point), the lack of post-treatment HRCT for over half of patients, the use of concomitant oral glucocorticoids and IS in all cases, and the lack of a control group. The last two issues made interpretation of RTX's effects on the natural course of ILD difficult. However, although the effects on lung progression could not be clearly attributed to RTX alone, in order to better account for this we included in our study only those patients with progressive ASS-ILD unresponsive to conventional therapy. In addition, ongoing treatment with IS remained unchanged in all patients during the post-RTX follow-up period. Patients served as their own controls (with pre- and post-treatment lung function trends) and provided convincing evidence of real treatment effects attributable to RTX rescue therapy, based on the fact that we used objective outcome measures independent on the examiner´s bias. Nonetheless, we cannot rule out other residual confounders affecting associations between RTX use and outcomes, since no adjusted analysis could be performed due to the small sample size.

However, our data reflects outcomes from realistic clinical practice in refractory cases involving this severe complication, with an additional strength being that our study is not part of a corporate-sponsored research initiative. In addition, patients were followed-up in a protocolized manner with standardized data collection, and the duration of exposure to rituximab was relatively long.

## Conclusion

Progressive ILD in the course of ASS significantly impacts the prognosis for patients and remains challenging to treat. Unfortunately, no treatment guidelines are currently available for patients with refractory ASS-ILD. Data from long-term observations are highly anticipated to evaluate the efficiency and safety of various therapeutic options. Limited data on RTX is available, particularly in cases with PPF.

Based on our results, RTX appears to be effective as a rescue therapy in most patients with recurrent or progressive ASS-ILD unresponsive to conventional treatment. Use of RTX was well tolerated in the majority of patients.

## Data Availability

All relevant data generated or analyzed during this study are included in this published article.

## Abbreviations

ARS: Anti-aminoacyl-transfer-RNA synthetases antibodies
ASS: Antisynthetase syndrome
ASS-ILD: Antisynthetase syndrome-related interstitial lung disease
ATS: American Thoracic Society
AZA: Azathioprine
CsA: Cyclosporine A
CTDs-ILD: Connective tissue disease-related interstitial lung disease
CYC: Cyclophosphamide
DM: dermatomyositis
ERS: European Respiratory Society
GC: Glucocorticoids
HCQ: Hydroxychloroquine
HRCT: Thoracic high-resolution computed tomography
ILD: Interstitial lung disease
IQR: Interquartile range
IS: Immunosuppressants
IV: Intravenous
MMF: mycophenolate mofetil
NSIP: nonspecific interstitial pneumonia
OP: organizing pneumonia
PFT: pulmonary function tests
PPF: progressive pulmonary fibrosis
%pFVC: predicted forced vital capacity
%pDLCO: predicted diffusing capacity for carbon monoxide, corrected for hemoglobin
RTX: Rituximab
6MWT: six-minute walking test
TAC: tacrolimus
