# Correction: Rituximab in the treatment of progressive interstitial lung disease associated with the antisynthetase syndrome

**DOI:** 10.1186/s13075-024-03357-y

**Published:** 2024-07-09

**Authors:** Javier Narvaez, Elena Canadillas, Ivan Castellvi, Juan Jose Alegre, Vanesa Vicens‑Zygmunt, Guadalupe Bermudo, Paola Vidal-Montal, Maria Molina Molina, Joan Miquel Nolla

**Affiliations:** 1 Department of Rheumatology, Hospital Universitario de Bellvitge. Bellvitge Biomedical Research Institute (IDIBELL), Feixa Llarga, s/n, Hospitalet de Llobregat, Barcelona, 08907 Spain; 2 Department of Rheumatology, Hospital Cl?nico, Universitario Lozano Blesa, Zaragoza, Spain; 3 Department of Rheumatology. Hospital, Universitario de la Santa Creu i Sant Pau, Barcelona, Spain; 4 Department of Rheumatology. Hospital, Universitario Dr Peset, Valencia, Spain; 5 Interstitial Lung Disease Unit, Department of Pneumology. Hospital, Universitario de Bellvitge. Bellvitge Biomedical Research Institute (IDIBELL), Barcelona, Spain

**Correction: Arthritis Res Ther 26, 122 (2024)**

10.1186/s13075-024-03353-2

Following publication of the original article [1], the authors identified an error in the author name of Vanesa Vicens‑Zygmunt.

The incorrect author name is: Vanesa Vincens‑Zygmunt.

The correct author name is: Vanesa Vicens‑Zygmunt.

The author group has been updated above and the original article [1] has been corrected.
